# Brigatinib treated ALK positive lung squamous cell carcinoma after failed chemotherapy: A case report

**DOI:** 10.1111/1759-7714.14133

**Published:** 2021-10-13

**Authors:** Shuluan Li, Pei Zhang, Tianyu Wang, Jie Wang, Jianchun Duan

**Affiliations:** ^1^ Department of Medical Oncology, National Cancer Center/National Clinical Research Center for Cancer/Cancer Hospital & Shenzhen Hospital Chinese Academy of Medical Sciences and Peking Union Medical College Shenzhen China; ^2^ Department of Medical Oncology National Cancer Center/National Clinical Research Center for Cancer/Cancer Hospital, Chinese Academy of Medical Sciences and Peking Union Medical College Beijing China; ^3^ Department of Breast Surgery National Cancer Center/National Clinical Research Center for Cancer/Cancer Hospital & Shenzhen Hospital, Chinese Academy of Medical Sciences and Peking Union Medical College Shenzhen China

**Keywords:** ALK, brigatinib, chemotherapy, lung squamous cell carcinoma

## Abstract

The definitive efficacy of anaplastic lymphoma kinase (ALK) inhibitors in ALK positive lung squamous cell carcinoma (sqCC) patients remain unclear. Here, we report a case in which brigatinib had a therapeutic effect on ALK‐positive lung squamous cell carcinoma. The patient in this report was diagnosed with ALK‐positive lung squamous cell carcinoma with brain metastases, and received brigatinib after failure of first‐line chemotherapy. Response duration was approximately 11 months, with tolerable side effects. In conclusion, a good clinical effect was achieved in a patient with ALK positive lung squamous cell carcinoma who received treatment with an ALK inhibitor.

## Graphical abstract

Our case was first report to determine the excellent antitumor effect of Brigatinib on ALK positive lung SqCC. PFS was more than 11 months, and the side effects were tolerable.

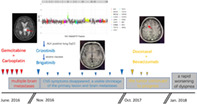

## INTRODUCTION

Reports of lung squamous cell carcinoma (SqCC) patients with anaplastic lymphoma kinase (ALK)‐rearrangement are truly rare.^1^, ^2^ Although ALK inhibitors have been reported to offer significant improvement in the clinical outcomes of patients with advanced ALK positive lung adenocarcinoma,^3^, ^4^, ^5^ there is a paucity of data on the efficacy of such treatment for ALK positive lung SqCC. Recently, brigatinib has become one of the preferred first‐line drugs used in ALK positive NSCLC patients. However, the efficacy of brigatinib in lung SqCC is still unknown. Here, we report a rare case of ALK positive lung SqCC in a patient who reacted well to brigatinib after failure of first‐line chemotherapy.

## CASE REPORT

**FIGURE 1:**
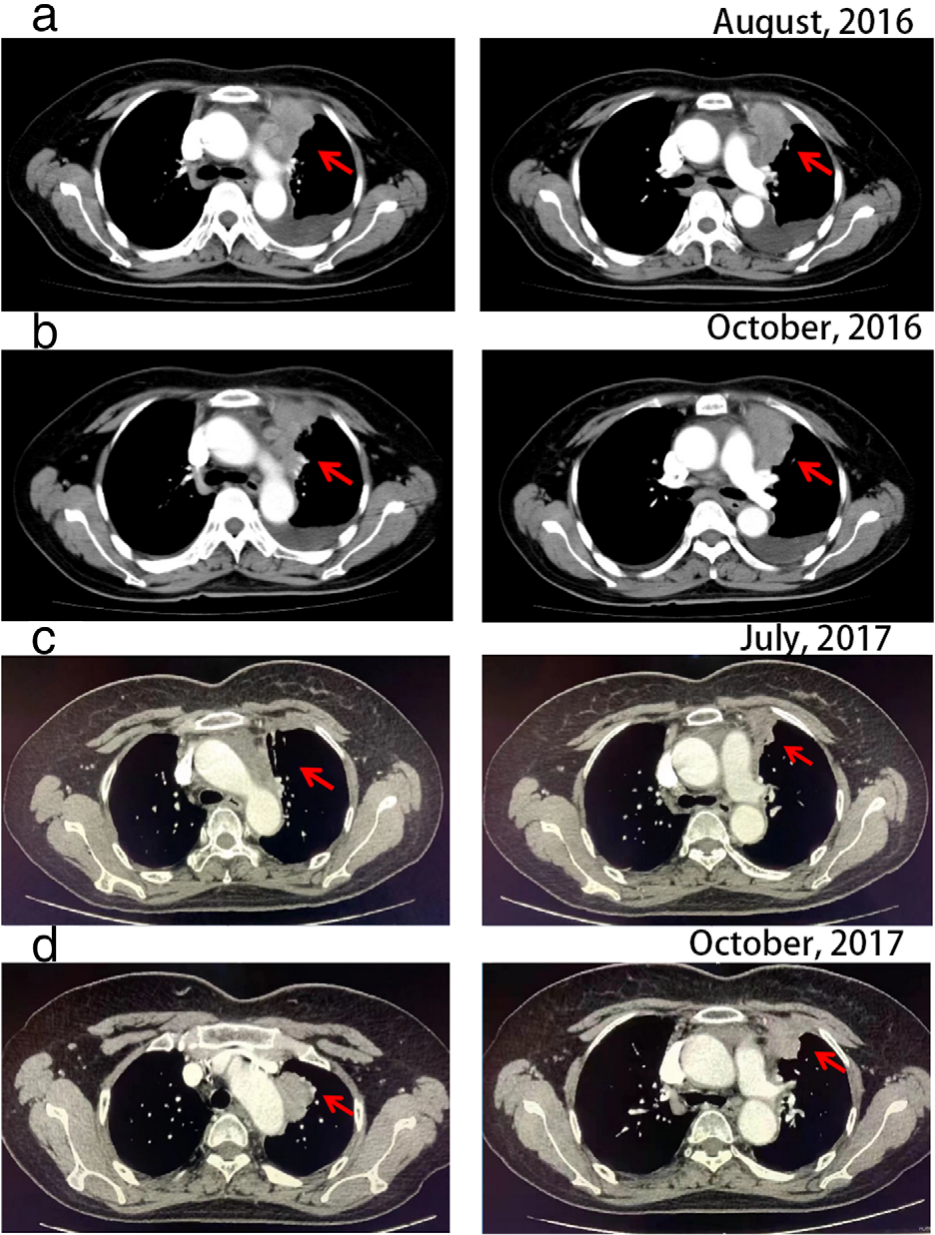
Computed tomography (CT) images of the patient's primary pulmonary tumor lesion (a) before chemotherapy (August 2016), (b) resistant to chemotherapy (October 2016), (c) response to brigatinib (July 2017), and (d) resistant to brigatinib (October 2017), respectively. The red arrows indicate the pulmonary lesion

In June 2016, a 49‐year‐old Chinese woman without a history of smoking history was referred to the hospital with chest suppression and a severe cough. Computed tomography (CT) of the chest located a primary lesion measuring 7.8 x 6.5 cm in the left superior lung lobe, with ipsilateral hilar and mediastinal lymphadenopathy, left pleural effusion and pericardial effusion (Figure 1a). Pathological examination of the supraclavicular lymph node biopsy revealed typical morphology of squamous cell carcinoma cells. Immunohistochemistry (IHC) analysis demonstrated that high molecular weight cytokeratin (CKH) in tumor cells was positive. Considering the small biopsy specimen and never‐smoker status, epidermal growth factor receptor (EGFR) and ALK assessment were requested. *EGFR* mutation was not detected. Fortunately, fluorescent in situ hybuidization (FISH) data showed ALK rearrangement. The rearrangement‐positive cell rate was 26.0%. According to these data, this patient was diagnosed with ALK positive lung SqCC (cT4N3M0).

Considering the standard first‐line treatment for lung SqCC is still platinum‐based dual‐drug chemotherapy,^6^, ^7^ at the same time, ALK rearrangement of this patient was performed through the small biopsy specimen. The patient was initially treated with gemcitabine plus carboplatin as first‐line chemotherapy (gemcitabine 1250 mg/m^2^ d1, d8; carboplatin AUC = 5 d2, per 21 days). After four cycles of chemotherapy, a follow‐up CT scan revealed stable disease (SD) response in the primary lesion (7.8 x 6.5 cm → 5.2 x 4.2 cm) (Figure 1b). However, the patient refused chemotherapy after five cycles due to adverse reactions (myelosuppression, fatigue and nausea). One month later, consciousness disorder appeared, and brain MR revealed multiple brain metastases (left thalamus mass 0.7 x 0.8 cm, frontal lobe lesion 0.4 x 0.8 cm). Second‐line therapy was therefore indicated.

**FIGURE 2:**
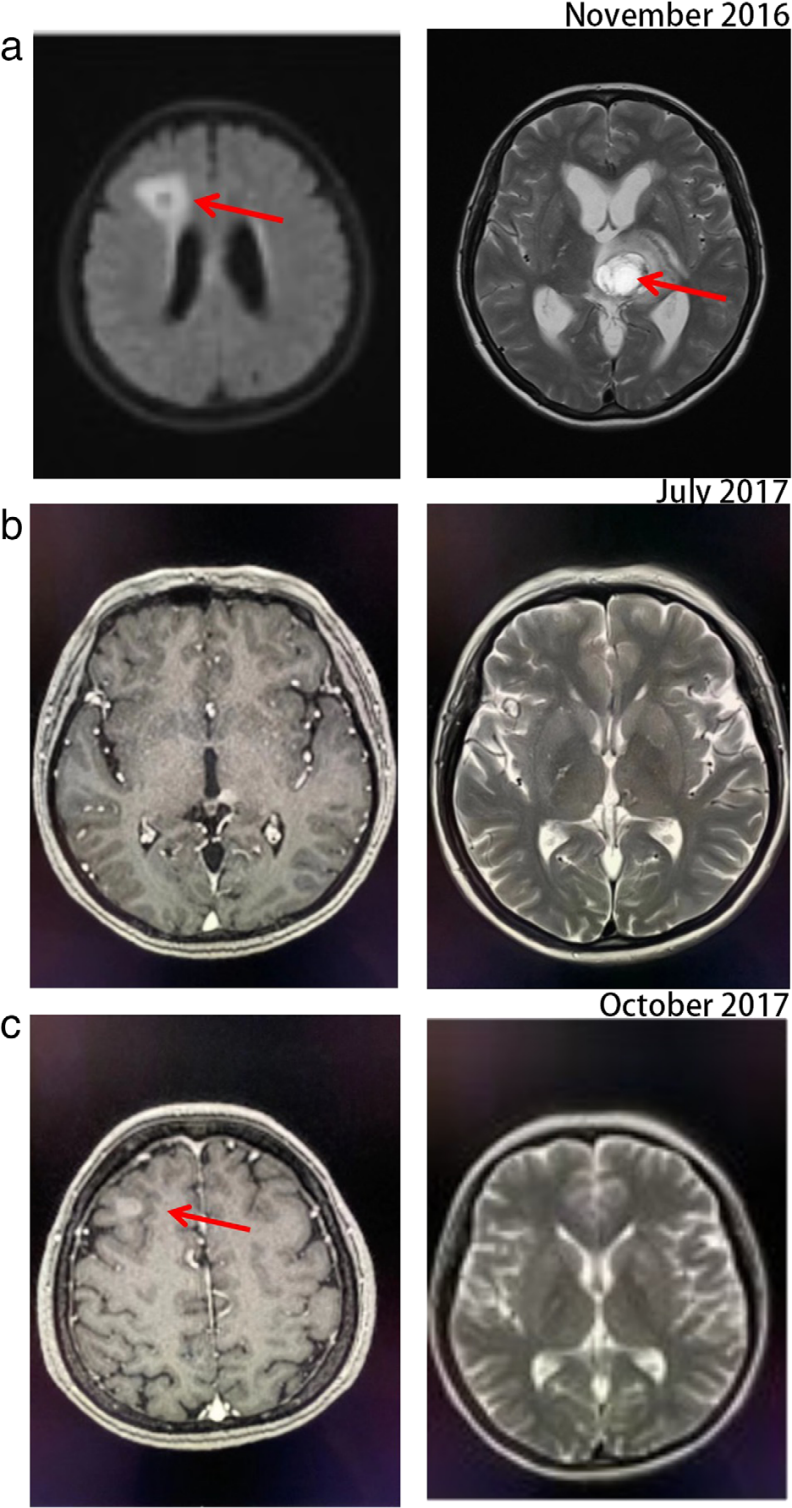
Magnetic resonance imaging (MRI) of the patient's metastatic brain tumor lesion (a) before brigatinib treatment (November 2016), (b) response to brigatinib (July 2017), and (c) resistant to brigatinib (October 2017). The red arrows indicate the brain lesion

In November 2016, considering the presence of the ALK gene rearrangement, crizotinib was prescribed as second‐line treatment. After one day of treatment the patient was changed from crizotinib to brigatinib 90 mg because of severe nausea. After one week of brigatinib treatment, the CNS symptoms surprisingly disappeared and the patient did not show any adverse events. Therefore, brigatinib was increased to 180 mg. One month later, a consecutive CT scan showed a visible shrinkage of the primary lesion (5.2 x 4.2 cm → 2.0 x 1.0 cm). Meanwhile, visible brain metastases were stable. In July 2017, CT scan re‐evaluation of the pulmonary and brain metastases (Figures 1c and 2) showed stable disease (SD). Moreover, the patient's side effects were tolerable.

**FIGURE 3:**
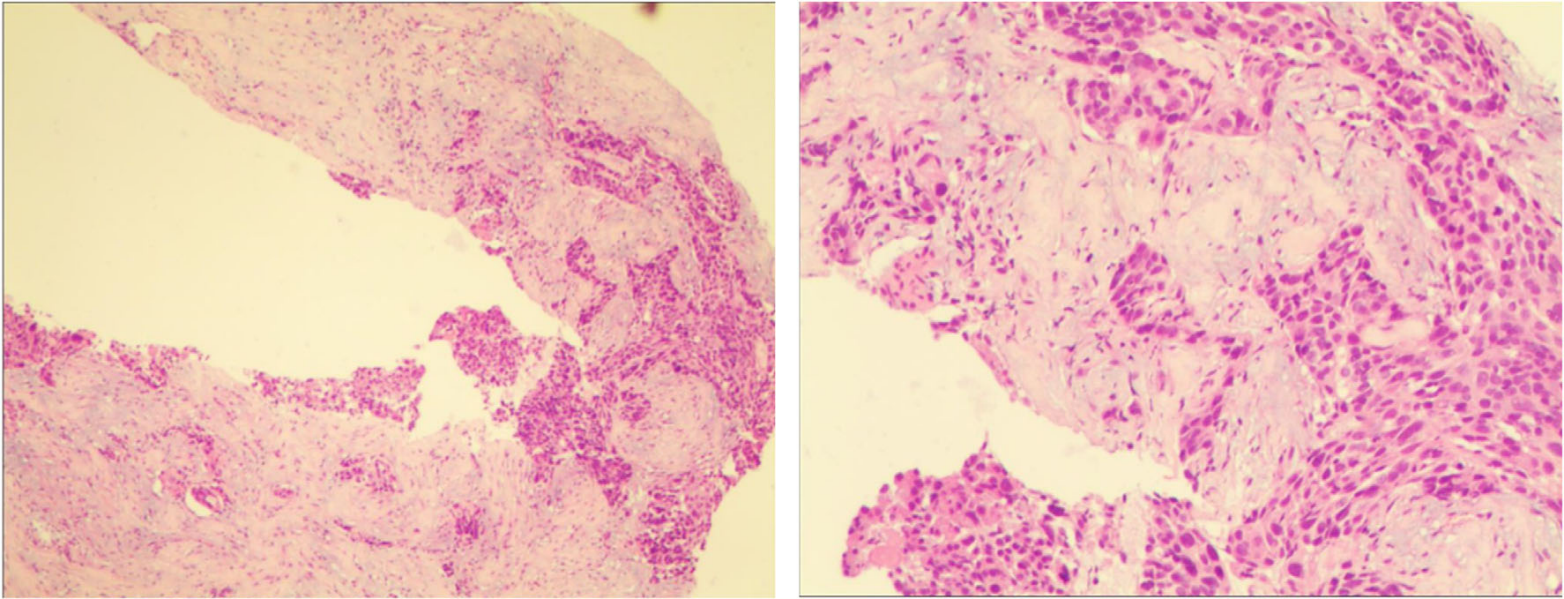
Computed tomography (CT)‐guided percutaneous transthoracic lung biopsy and immunohistochemistry (IHC) analysis of the biopsy (November 2017): P40(+), CK(+), CD56(−), TTF‐1(−), Ki‐67 (30%+), ALK (3+)

Brigatinib treatment continued for another 3 months but in October 2017 the CT scan re‐evaluation showed lung lesion progression and a new metastatic brain lesion (Figures 1d and 2). CT‐guided percutaneous transthoracic lung biopsy was performed and a SqCC diagnosis was again histologically and immunohistochemically confirmed. IHC analysis of the biopsy demonstrated that tumor cells were positive for P40 and cytokeratin (CK), and negative for CD56 and thyroid transcription factor‐1 (TTF‐1). The positive rate of Ki‐67 was almost 30%. ALK was strongly expressed in this case (3+ staining) (Figure 3). Next‐generation sequencing (NGS) including 56 gene mutations, rearrangements, copy number changes, or single nucleotide polymorphisms highly related to lung cancer showed that the abundance of EMLA4‐ALK translocation was 7.28%. Because of tumor progression, our patient changed to chemotherapy (docetaxel 75 mg/m^2^, per 21 days). Unfortunately, there was still tumor progression.

In January 2018, the patient experienced a rapid worsening of dyspnea and began receiving best supportive care. One month later the patient died.

## DISCUSSION

So far, there have only been a few cases reported in which ALK rearrangements in pure SqCC have been identified together with their response to ALK inhibitors.^8^, ^9^, ^10^, ^11^, ^12^, ^13^, ^14^, ^15^, ^16^ Our study is the first report to determine the excellent antitumor effect of brigatinib on ALK positive lung SqCC. Progression‐free survival (PFS) in the patient in our study was more than 11 months, with tolerable side‐effects.

A next‐generation ALK inhibitor, brigatinib (AP26113) has been reported to have robust efficacy in patients with ALK gene rearrangement NSCLC that is refractory to crizotinib.^17^, ^18^, ^19^ The Food and Drug Administration (FDA) have granted approval for brigatinib to be used in the first‐line treatment of patients with ALK‐positive NSCLC. Moreover, brigatinib is an important ALK inhibitor for the effective treatment of CNS disease. Camidge et al. has previously reported that the intracranial response rate of the brigatinib group in their study was 78% (95% CI: 52–94).^20^ In this case, the patient was diagnosed with squamous cell carcinoma twice by pathological immunohistochemistry, and both immunohistochemistry and second‐generation sequencing demonstrated the presence of ALK gene rearrangement. The patient exhibited a high antitumor response to brigatinib, both in CNS disease and extra‐CNS disease. Therefore, brigatinib might be another appropriate TKI for patients with ALK‐rearranged lung SqCC, especially in patients with CNS disease.

In conclusion, an ALK inhibitor might be the most appropriate treatment in SqCC patients with ALK rearrangement, especially patients who are both ALK IHC positive and confirmed to have a high frequency of neoplastic nuclei by FISH testing.

## CONFLICT OF INTEREST

The authors declare no conflict of interest.
